# Orthopaedic Surgery Residency: Perspective of Applicants and Program Directors on Medical Student Virtual Experiences

**DOI:** 10.5435/JAAOSGlobal-D-22-00278

**Published:** 2023-04-05

**Authors:** Justin J. Hicks, Youssef M. Khalafallah, Joshua Wright-Chisem, Mary K. Mulcahey, William N. Levine, Dawn LaPorte, Joshua Patt, Monica Kogan

**Affiliations:** From the Washington University of St. Louis Orthopaedics Department, St. Louis, MO (Dr. Hicks); the Department of Orthopaedic Surgery, Baylor College of Medicine, Houston, TX (Dr. Khalafallah); the Department of Orthopaedic Surgery, Hospital for Special Surgery, New York, NY (Dr. Wright-Chisem); the Department of Orthopaedic Surgery, Tulane University School of Medicine, New Orleans, LA (Dr. Mulcahey); the Department of Orthopedic Surgery, Columbia University Medical Center, New York, NY (Dr. Levine); the Department of Orthopaedic Surgery, The Johns Hopkins University School of Medicine, Baltimore, Maryland (Dr. LaPorte); the Department of Orthopedic Surgery Atrium Musculoskeletal Institute, Charlotte, NC (Dr. Patt); and the Department of Orthopedic Surgery, Rush University Medical Center, Chicago, IL (Dr. Kogan).

## Abstract

**Introduction**: Orthopaedic Residency Directors advised against visiting subinternship rotations in the peak of the pandemic. To adapt, programs offered multiple virtual experiences. The purpose of this study was to evaluate programs and applicants perceptions regarding the value of virtual experiences during the 2020 to 2021 application cycle and their utility in future application cycles.

**Methods**: A survey was disseminated to 31 residency programs gathering data about virtual experiences offered in this cycle. A second survey was disseminated to interns who have successfully matched at those programs to identify how interns think to have benefited from the experiences.

**Results**: Twenty-eight programs completed the survey (90% response rate). One hundred eight new interns completed the survey (70% response rate). Virtual information sessions and resident socials were the highest attended (94% and 92%, respectively). Interns and leadership agreed that virtual rotations provided students with a good understanding of program culture and what the programs offer educationally. Neither the leadership nor the interns would recommend replacing in-person aways with virtual experiences.

**Conclusion**: Virtual experiences helped bridge the gap after away rotations were canceled. Alongside in-person aways, virtual experiences are likely to play a role in future cycles. However, virtual experiences remain incomparable to in-person away rotations and are not recommended as a replacement.

Orthopaedic surgery remains one of the most competitive and sought after medical or surgical residencies. In 2015, there were 1,062 applicants pursuing 703 residency spots, with graduates of US medical schools applying to an average of 70 of 161 residency programs.^1^ Orthopaedic surgery residency programs on average received 572 applications for 4.4 available intern positions and filled 100% of them through the match process. There has been application inflation with 1,289 US medical school applicants for 868 positions in 2021, with less than 1% of available positions going unfilled over the past 30 years.^2,3,4,5^ Orthopaedic surgery applicants are consistently among the top applicants for all medical and surgical specialties regarding class rank, United States Medical Licensing Examination (USLME) step 1 scores, Alpha Omega Alpha status, and research productivity.^6^ Given the competitiveness of matching into orthopaedic surgery, the visiting orthopaedic surgery rotation (also referred to as externship, subinternship, away, or away rotation) remains one of the most important aspects of the application process.^7,8^ Cohn et al,^9^ in a survey of orthopaedic surgery program directors, found student subinternship performance to be the single most important factor in resident selection. With USMLE step 1 becoming pass/fail, it is thought that even more weight will be placed on the subinternship performance.^9^

In the wake of the COVID-19 pandemic, the Council of Orthopaedic Residency Directors (CORD) advised against visiting subinternship rotations. Exceptions were made for students without a home orthopaedic surgery program (“orphan program”) or where an away rotation was a graduation requirement. These new recommendations raised notable concerns for applicants and program directors alike. Without in-person subinternship experiences, there was a fear that programs would be unable to adequately assess how applicants would integrate in their respective programs. Furthermore, there were concerns that applicants may not gain the benefits of away rotations, some of which include assessing the environment of a prospective program, establishing professional relationships, obtaining letters of recommendation, and furthering orthopaedic education, mentorship, as well as an opportunity to show one's work ethic or ability to work in a team. To adapt and abide by CORD's recommendations, many orthopaedic surgery programs offered virtual experiences. The goal was for these virtual experiences to ideally provide the same benefits to both applicants and programs afforded by in-person away rotations.

The purpose of this study was to ascertain program leaderships' and applicants' perceptions about the value of the virtual experiences during the 2020 to 2021 application cycle and the utility of different virtual experiences in future application cycles.

## Methods

### Survey Construction

Two surveys were built in the Qualtrics online platform (Qualtrics) (Appendix 1, https://jhmi.co1.qualtrics.com/jfe/form/SV_8G4ogCmz7AxtLTg and https://qfreeaccountssjc1.az1.qualtrics.com/jfe/form/SV_aWxLaHsRdBlR9NY). A 10-question online survey was designed to target residency programs' leadership gathering data about virtual electives offered during the 2020 to 2021 application cycle at their programs. Leadership was surveyed about the selection process for applicants offered to participate in their virtual opportunities and how successful those different virtual opportunities were at portraying the different aspects of their program. A second eight-question survey was designed to target the interns of class of 2026 to identify the virtual opportunities they had participated in during their 2020 to 2021 application cycle. The surveys were piloted and reviewed by the study team, which included both faculty and residents. In an effort to increase the response rate, surveys were anonymous. In addition, respondents were allowed to freely response to further clarify or expand on any of their answers.

### Survey Dissemination

The leadership survey was disseminated to the 31 residency programs who had expressed interest in participation during the Collaborative Orthopaedic Educational Research Group monthly meeting. A follow-up email was sent 3 weeks later. The resident survey was shared with the leadership of those same 31 programs and was disseminated by the respective leadership to their incoming interns.

### Data Collection and Statistical Analysis

Survey responses were recorded by Qualtrics and exported into Microsoft Excel. Descriptive analysis and graphic representation were used to report categorical responses about virtual opportunities, and their presumed benefit to the students, and programs.

## Results

For the leadership survey, 28 programs took the survey (90% response rate, with some incomplete responses). Most of the respondents were program directors (21 PDs), but 6 associate PDs and one chairperson participated as well. During the 2020 to 2021 cycle, most programs did not allow any in-person away rotations for various reasons (Table 1). However, three programs (11%) made exemptions for students from orphan programs. To keep the remaining applicant pool and those not from the orphan programs informed about what various programs had to offer, these cycle programs expanded in the virtual opportunities. Eighty-two percent of the programs (N = 23) surveyed offered virtual information sessions, and 43 percent of the programs (N = 12) developed longitudinal virtual curricula/rotations (Table 2).

**Table 1:** Reasons Why Programs Did Not Offer an Away Rotation

Reasoning	Count
Thought it would be challenging and unfair for some students given their medical school commitments and wanted to be equitable	11
Institutional policy	6
Hosted away rotations for orphan programs	3
Offered to orphan students but only own students rotated with them	2
Thought it would be difficult to coordinate	2
Thought there would not have been support from attendings/residents to help	1
Thought this would not be helpful for students or the program	0

**Table 2:** Various Types of Virtual Opportunities Offered in This Cycle

Opportunity Description	Count
Students participated in a virtual away rotation that lasted 2 wk, with mandatory attendance, where students were given an opportunity to present and demonstrate their orthopaedic knowledge and skills	1
Students participated in a virtual away rotation that lasted 4 wk, with mandatory attendance, where students were given opportunities to present and demonstrate their orthopaedic knowledge and/or skills.	0
Students participated in a virtual experience with optional attendance where students were given opportunities to present and demonstrate their orthopaedic knowledge or skills.	4
Students participated in a virtual experience with optional experience where students did not formally present or demonstrate their knowledge or skills	6
The program hosted virtual informational meetings	23
The program hosted virtual resident socials	7
The program hosted virtual events specifically for underrepresented minorities or women	1
Other: longitudinal program for 6 mo	1

The selection process, expectations, and opportunity to demonstrate knowledge with virtual rotations seem to be different from in-person away rotations. Most of the programs were nonselective about accepting all students who expressed interest, had no expectation of mandatory attendance, and lacked opportunities for students to demonstrate their orthopaedic knowledge and skill. However, programs varied. Two programs only accepted those who had initially applied for an in-person away rotation or used an additional application, and one program mandated attendance for 2 weeks and offered an opportunity for students to demonstrate their knowledge (Tables 2 and 3). Fifty-four percent of the program leaders (N = 6) think that students were able to participate in the virtual opportunities without conflict (Table 4).

**Table 3:** How Students Were Selected to Participate in Virtual Opportunities

Selection process	Count
There was an application specifically for a formal virtual experience.	3
It was offered only to students who had already applied for an in-person away rotation.	2
It was offered to any student who wanted to participate.	6

**Table 4:** Leadership's Rating and Opinion About the Utility of Virtual Rotations and Experiences

Opinion	Strongly Disagree	Disagree	Neutral	Agree	Strongly Agree
After the virtual experience, most students felt like they had a good understanding of what the program had to offer educationally.	—	—	18%	55%	27%
After the virtual experience, most students felt like they had a good understanding of what the program had to offer surgically.	9%	9%	36%	18%	18%
The virtual away/experience allowed most students to get a feel for the culture of the program.	9%		18%	45%	27%
Most students were able to get a feel of what the residents were like during the virtual experience.	9%	9%	9%	36%	36%
Most students were able to get a feel of resident/fellow interactions during the virtual experience.	9%	18%	27%	27%	18%
The virtual away/experience allowed students to demonstrate their strengths as an applicant to the program.	9%	45%	9%	36%	
The virtual away/experience allowed students to form good relationships and gain strong mentorships from faculty participants.	—	55%	18%	18%	9%
The students who attended the away rotations/experience were able to do so without conflicts from medical school responsibilities.	—	27%	18%	18%	36%
Would recommend virtual aways/experiences as an option for the future in place of an in-person rotation.	18%	36%	18%	9%	18%

Residency program leaders agreed about certain aspects of the virtual experiences. Eighty-two percent of the program leaders (N = 9) either agreed or strongly agreed that the virtual experiences led to a good understanding of what their program had to offer educationally. Seventy-two percent (N = 8) agreed or strongly agreed, while 18% (N = 2) disagreed or strongly disagreed that these experiences allowed students to get a feel for the culture of their program. Seventy-two percent of the leaders (N = 8) agreed or strongly agreed that virtual experiences permitted applicants to get a feel of what their residents were like (Table 4). Conversely, programs, including those that have deliberately offered an opportunity for students to present and demonstrate their knowledge, do not think that their virtual experience allowed the students an adequate opportunity to demonstrate their strengths (54% [N = 6] disagreed or strongly disagreed) nor an opportunity to form strong relationships with faculty and mentors (55% [N = 6] disagreed and 18% [N = 2] neither agreed nor disagreed). Opinions varied regarding insight into what the programs had to offer surgically as well as the dynamic between the residents and the fellows at their program (Table 4). Overall, only 27% of the program leaders would recommend virtual experiences in place of in-person away rotations versus 54% who would not make this recommendation (Table 4).

For the intern survey, 108 of 156 interns took the survey (70% response rate with some incomplete responses). Seventy-four percent of the respondents were male, 24% female, and 2% other. Sixty-two percent of the interns surveyed were White, 9% Black or African American, 6% Hispanic/Latin or of other Spanish origin, 18% Asian, 2% Other, and 4% preferred not to respond (Table 5). All but one intern participated in virtual experiences during their application cycle (Table 6). Virtual information sessions and resident socials were the highest attended (94% and 92%, respectively). Regarding the utility of different opportunities, information sessions, longitudinal optional rotations, and mandatory rotations all were rated to be beneficial by a roughly equal proportion of interns who completed those opportunities (65%, 63%, and 64%, respectively). Resident socials had a slightly lower proportion of those who rated it to be helpful (55%), yet it remains to be a majority (Table 6).

**Table 5:** Resident Participants' Demographics

Answer	Count (%)
Sex	
Female	26 (24%)
Male	80 (74%)
Other	2 (2%)
I prefer not to respond	0
Race/ethnicity	
White	66 (62%)
Black or African American	10 (9%)
Hispanic, LatinX, or of other Spanish origin	6 (6%)
Asian	19 (18%)
Other	2 (2%)
I prefer not to respond	4 (4%)

**Table 6:** Types of Virtual Experiences and Rotations That Interns Have Participated in During Their Application Cycle

Types of Virtual Experiences/Rotations	Participation (%)	Helpful (%)^a^	Not Helpful (%)^a^
Virtual informational meetings	102 (94%)	66 (65%)	8 (8%)
Resident socials	99 (92%)	54 (55%)	14 (14%)
Virtual experience where participation was optional	73 (68%)	46 (63%)	17 (23%)
Formal virtual away rotation that lasted 4 wks where attendance was mandatory	36 (33%)	23 (64%)	21 (58%)
Did not participate in any virtual experience	1 (1%)	—	—

a Percentages were calculated based on the number of participants who have completed this type of virtual experience from the participation column.

**Figure 1:**
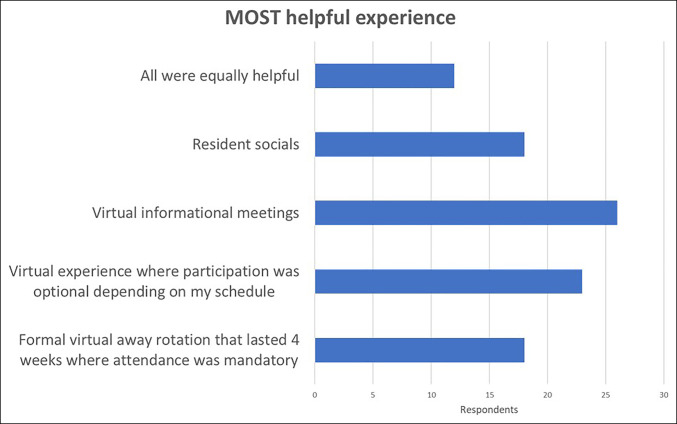
Graphic representation of the distribution of the experience that the interns rated to have been the “MOST Helpful” experience.

Longitudinal mandatory sessions seemed to be slightly controversial because they were the most frequently cited opportunity to be “THE MOST helpful” but also the most frequently cited by interns to “NOT find helpful.” However, of the 21 interns who rated this opportunity “not helpful,” 13 interns had not actually participated in those sessions (Figure 1).

Many of the interns shared a similar opinion with the leadership about virtual rotations allowing them a good understanding of what the programs had to offer educationally (76% [N = 75] agreed or strongly agreed), what the residents are like (63% [N = 62] agree or strongly agree), and what the culture of the program is (63% [N = 62] agreed or strongly agreed) (Supplemental Table, http://links.lww.com/JG9/A257). 62% of the interns also agreed/strongly agreed that the virtual rotations lacked the opportunity for them to demonstrate their strengths. Sixty percent of the interns (N = 60) felt that virtual experiences lacked the opportunity to form strong relationships with faculty and mentors. Opinions varied about the interns' insight into what the programs had to offer surgically (Supplemental Table, http://links.lww.com/JG9/A257). Fifty-two percent of the interns (N = 52) thought that virtual experiences allowed them to gain an understanding of the dynamic between the residents and the fellows. Almost an equal proportion of interns reported conflicts with the medical school responsibilities compared with those who denied conflicts (39% [N = 37] versus 41% [N = 39]). Finally, 70% of the interns [N = 68] would not recommend virtual experiences to replace in-person rotations in the future. Only 2% of the interns (N = 2) would prefer a virtual experience rather than an in-person away rotation, while 64% of the interns (N = 60) thought that they would have had better chances of matching at the program of their choice had they completed in-person away rotations this past cycle (Supplemental Table, http://links.lww.com/JG9/A257).

## Discussion

Matching into orthopaedic surgery is highly competitive, and the rate of unmatched applicants has remained relatively unchanged since 1984.^3^ Previous studies have analyzed characteristics and strategies to successfully navigate the orthopaedic surgery match. Most applicants have similar application profiles including class rank, high USLME step 1 scores, Alpha Omega Alpha status, and publication volume. Previous studies have demonstrated that the subinternship is considered to be a critical component of the application to help distinguish applicants with similar study characteristics. In 2002, the most important factor for resident selection was performance on orthopaedic subinternships.^10^ A recent study demonstrates that 18 years later, this sentiment remains.^9^ There are benefits of away rotations from both the program perspective and the applicant perspective. Away rotations are essentially a 4-week interview or audition where residency programs can assess how successfully a student will integrate into their program.^11^ For students, the away rotation serves as a way to assess the culture of a prospective program, evaluate the educational curriculum, and see the surgical case volume. It can also serve as a way for students to build strong professional relationships or obtain a letter of recommendation from a physician outside of their home program.^11^ This can be especially valuable for orphan students, students from smaller nonacademic programs, and students underrepresented in orthopaedic surgery. For the orphan program applicants, the away rotation becomes even more critical, allowing students to gain mentorship that they might not have available at their home institution or afford them an opportunity to be educated in orthopaedic surgery. For female or underrepresented minority students, it may also allow them to see whether the culture of the program is one that would be welcoming. In this study, both applicants and PDs agreed that the virtual environment did not allow students to form strong professional relationships with faculty. Applicants also expressed difficulty in demonstrating their strengths to residency programs and thought that they would have a better chance of matching had they been able to participate in an in-person away rotation. Only 2% of interns indicated that they would prefer a virtual away experience to an in-person away rotation.

During away rotations, students are fully immersed in the away rotation environment and excused from any obligations with their home institutions. This was often not the case for students participating in virtual opportunities. Applicants were commonly not granted school credit for these virtual experiences and were, therefore, completing virtual experiences around other educational requirements, making the process cumbersome. Eighty-two percent of the programs (N = 23) surveyed offered virtual information sessions, and 43% of the programs (N = 12) developed virtual curricula/rotations. These longitudinal experiences were quite variable lasting between 2 weeks and 6 months, half of which did not give students the opportunity to present or demonstrate orthopaedic knowledge (Table 2). The programs seem to have underestimated the ability of students to attend and participate in virtual experiences without difficulty. Fifty-four percent of the program leaders thought that students were able to participate in the virtual opportunities without conflict, while only 32% of the interns agreed and 9% of the interns strongly agreed they were able to participate without conflicts. The mandatory experiences that lasted 4 weeks or more were anecdotally most difficult for students to schedule around their core medical school requirements.

For in-person away rotations, notable heterogeneity exists for both the selection of students and the organizational structure of the rotation.^12^ Similarly, we found variability in the types of virtual opportunities offered by programs and how students were offered and/or selected for participation. More work is needed to determine the optimal virtual experience. Optional experiences and informational meetings were found to be most helpful.

The strength of our study includes high response rates by both program leadership and applicants on a topic that remains important to the orthopaedic application cycle. This is especially true because the Coalition for Physician Accountability's work group on away rotations discusses the future of in-person away rotations. In-person away rotations may become even more critical as we transition into an era of pass/fail grading for the USLME step 1. Program leadership and interns felt that virtual experiences should not be used in place of in-person away rotations. Recall bias is always a concern with retrospective survey studies. Our response rate and demographic representation of respondents limit bias in our study. Finally, our survey was only disseminated among successfully matched applicants, and our study lacks data from unmatched applicants.

## Conclusions

Virtual opportunities allowed students to get a good sense about the prospective program's culture and educational structure. Virtual information sessions and virtual experiences where participation was optional were found to be most helpful. Applicants found that it was difficult to demonstrate their strengths to residency programs or form strong relationships with faculty and thought that they would have a better chance of matching had they been able to participate in in-person away rotations. There may be potential for hybrid experiences in the future; however, virtual opportunities remain incomparable with what in-person rotations had offered and are not recommended to substitute for them.

## References

[1] National Resident Matching Program: Results and Data: 2015 Main Residency Match®.

[2] National Resident Matching Program: Results and Data: 2021 Main Residency Match®.

[3] Karnes JM Mayerson JL Scharschmidt TJ: Is orthopedics more competitive today than when my attending matched? An analysis of national resident matching program data for orthopedic PGY1 applicants from 1984 to 2011. J Surg Educ 2014;71:530-542. 24836166 10.1016/j.jsurg.2014.01.003

[4] Trikha R Keswani A Ishmael CR Greig D Kelley BV Bernthal NM: Current trends in orthopaedic surgery residency applications and match rates. J Bone Joint Surg Am 2020;102:e24. 31904608 10.2106/JBJS.19.00930

[5] Nasreddine AY Gallo R: Applying to orthopaedic residency and matching rates: Analysis and review of the past 25 years. J Bone Joint Surg Am 2019;101:e134. 31567661 10.2106/JBJS.18.00371

[6] O'Donnell SW Drolet BC Brower JP LaPorte D Eberson CP: Orthopaedic surgery residency: Perspectives of applicants and program directors on medical student away rotations. J Am Acad Orthop Surg 2017;25:61-68. 28002215 10.5435/JAAOS-D-16-00099

[7] Camp CL Sousa PL Hanssen AD : The cost of getting into orthopedic residency: Analysis of applicant demographics, expenditures, and the value of away rotations. J Surg Educ 2016;73:886-891. 27184179 10.1016/j.jsurg.2016.04.003

[8] Camp CL Wang D Turner NS Grawe BM Kogan M Kelly AM: Objective predictors of grit, self-control, and conscientiousness in orthopaedic surgery residency applicants. J Am Acad Orthop Surg 2019;27:e227-e234. 30247313 10.5435/JAAOS-D-17-00545

[9] Cohn MR Bigach SD Bernstein DN : Resident selection in the wake of United States medical licensing examination step 1 transition to pass/fail scoring. J Am Acad Orthop Surg 2020;28:865-873. 32925383 10.5435/JAAOS-D-20-00359

[10] Bernstein AD Jazrawi LM Elbeshbeshy B Valle CJD Zuckerman JD: Orthopaedic resident-selection criteria. J Bone Joint Surg Am 2002;84:2090-2096. 12429773 10.2106/00004623-200211000-00026

[11] Porter SE Jobin CM Lynch TS Levine WN: Survival guide for the orthopaedic surgery match. J Am Acad Orthop Surg 2017;25:403-410. 28489710 10.5435/JAAOS-D-17-00196

[12] Blood T Hill K Brown S Mulcahey MK Eberson CP: Variability of the orthopaedic away rotation: A survey of orthopaedic program directors. J Am Acad Orthop Surg Glob Res Rev 2021;5:e21.00024. 10.5435/JAAOSGlobal-D-21-00024 PMC7954370 33720055

